# May-Thurner Syndrome Variant Identified in a Cadaver: First Reported Case

**DOI:** 10.7759/cureus.2396

**Published:** 2018-03-30

**Authors:** Emily Simonds, Mayank Patel, Marc Vetter, Joe Iwanaga, Rod J Oskouian, R. Shane Tubbs

**Affiliations:** 1 Seattle Science Foundation; 2 Clinical Anatomy Research, Seattle Science Foundation; 3 Neurosurgery, Swedish Neuroscience Institute; 4 Neurosurgery, Seattle Science Foundation

**Keywords:** may-thurner syndrome, cockett syndrome, iliac vein compression syndrome, iliocaval compression syndrome, common iliac artery, plaques, artherosclerotic

## Abstract

May–Thurner syndrome (MTS) is defined as the compression of the left common iliac vein by the right common iliac artery. Herein, we describe an unusual case of a male cadaver with right-sided compression of the inferior vena cava and the left and right common iliac veins by the right common iliac artery. This is an unusual variant of this syndrome and the first known case report. We suggest this variant be termed MTS type II due to the additional compression of the inferior vena cava.

## Introduction

May-Thurner syndrome (MTS) is a rare disorder defined as obstruction caused by chronic left common iliac vein compression by the overlying right common iliac artery [1]. MTS is also known as iliac vein compression syndrome and is one of several pathologies included in the disease profiles known as nonthrombic iliac vein lesions. Such compression of the left common iliac vein by the overlying right common iliac artery inhibits venous blood return from the lower extremity and pelvis [1]. Variants of MTS include isolated compression of the right iliac vein or inferior vena cava (IVC) [2-3]. We present an unusual variant of MTS where the inferior vena cava and left and right common iliac veins were all compressed by an atherosclerotic right common iliac artery in a cadaver.

## Case presentation

**Figure 1:**
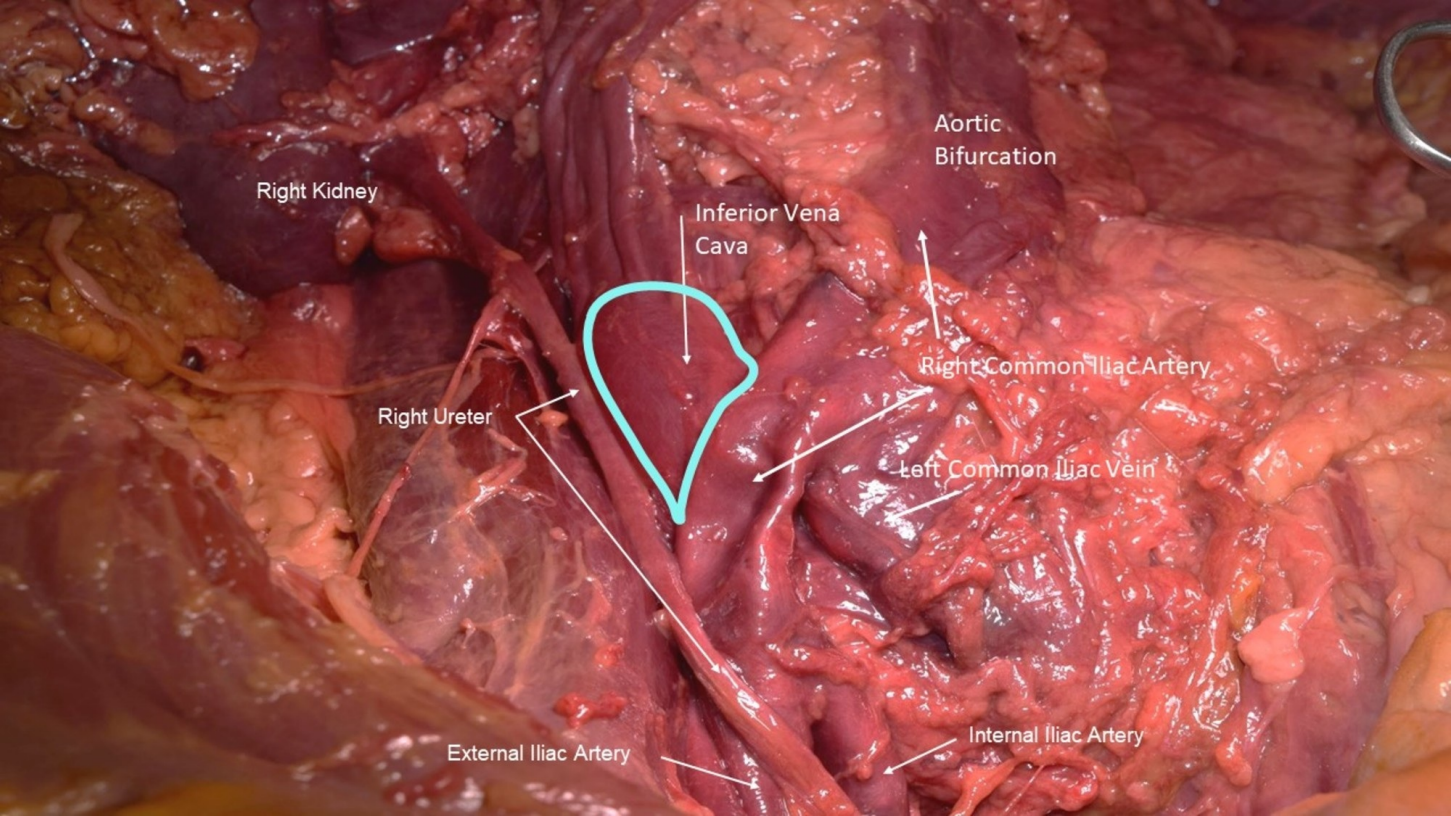
Anterior dissection of male cadaver with MTS variant The right common iliac artery is shown crossing over the anterior surface and compressing the distal inferior vena cava and left common iliac vein. The right common iliac vein (not shown) is also compressed. Note the dilated distal part (outlined in teal) of the inferior vena cava. Also, note the dimpling of the right common iliac artery due to atherosclerotic plaque within this vessel. The left common iliac artery is not shown.

During the routine dissection of the abdominopelvic region of a fresh frozen African American male cadaver (74 years old at death), the right common iliac artery was observed to be atherosclerotic and deviated more superiorly than normal (Figure 1). This resulted in simultaneous compression of the distal inferior vena cava and the left and right common iliac veins. The aorta bifurcated at the L4 vertebra and the inferior vena cava bifurcated at the L5 vertebral body. In the supine position, venous stasis was obvious in the prestenotic part of the inferior vena cava (Figure 1). Digital pressure of this engorged segment was not sufficient to propel venous blood within the inferior vena cava distally. Blood flow was only possible with anterior displacement of the overlying and atherosclerotic right common iliac artery, thus illustrating the compressive nature of the overlying right common iliac artery. No obvious signs of lower limb or pelvic visceral edema were noted. The diagnosis of an MTS variant was made, i.e., not only was the left common iliac vein compressed, but also the distal inferior vena cava and right common iliac vein.

## Discussion

To our knowledge, this is the first known case report of a MTS variant in a cadaver. Patients with MTS may present with discomfort in their left lower extremities due to pain, swelling, and deep vein thrombosis (DVT) [1]. One of the limitations of this case report is that the presence of these clinical symptoms are unknown in our specimen. However, the findings from our dissection are indicative of MTS. Pain from venous claudication after exercise occurs in 85% of symptomatic MTS patients [4].

The prevalence of this disease is also unknown, but it is estimated that 2-5% of all lower extremity venous disease can be attributed to MTS, which occurs more often in women than men [1]. MTS often goes undiagnosed and may only become symptomatic when there is trauma, or a significant surgery such as knee or hip replacement is involved. There are several rare presentations which can occur such as acquired MTS after endovascular stent/graft procedures [5-6]. MTS may present secondary to urinary bladder distention [7]. MTS may also play a role in spontaneous left iliac vein rupture due to spontaneous retroperitoneal hematoma in women with lower extremity DVT [8].

Clinical management of MTS includes compression stockings, evaluations by vascular surgeons, cardiology, and interventional radiology. Endovascular stenting of the iliac veins or open repair may be necessary [9].

## Conclusions

The case reported herein is unusual. To our knowledge, this is the first case of such a variant identified in a cadaveric specimen. We suggest this variant be termed MTS type II due to the additional compression of the inferior vena cava. Clinicians must consider MTS as a possible diagnosis when patients present with acute pain or swelling in the lower extremities.
